# Natural presence of the V179D and K103R/V179D mutations associated with resistance to nonnucleoside reverse transcriptase inhibitors in HIV-1 CRF65_cpx strains

**DOI:** 10.1186/s12879-020-05007-5

**Published:** 2020-04-28

**Authors:** Yongjian Liu, Yu Zhang, Hanping Li, Xiaolin Wang, Lei Jia, Jingwan Han, Tianyi Li, Jingyun Li, Lin Li

**Affiliations:** Department of AIDS Research, State Key Laboratory of Pathogen and Biosecurity, Beijing Institute of Microbiology and Epidemiology, Beijing, 100071 China

**Keywords:** HIV, CRF65_cpx, V179D, Drug resistance

## Abstract

**Background:**

There is increasing evidence that HIV-1 genetic diversity can have an impact on drug resistance. The aim of this study is to investigate the epidemiological situation of CRF65_cpx and the impact of natural polymorphisms of this variant on genotypic resistance.

**Methods:**

We used the BLAST search program followed by phylogenetic analysis to identify additional CRF65_cpx *pol* sequences from the Los Alamos HIV Sequence Database. Maximum likelihood phylogeny was estimated to clarify the epidemiological relationship of CRF65_cpx strains. Genotypic resistance was determined by submitting sequences to the Stanford HIV Drug Resistance Database.

**Results:**

A total of 32 CRF65_cpx *pol* sequences were obtained. The CRF65_cpx strains were detected in seven provinces with large geographic distance. Yunnan CRF65_cpx sequences were mainly derived from a heterosexual risk group, whereas the CRF65_cpx sequences in other provinces were almost exclusively derived from an MSM population. With one exception of V179E, the other 31 strains harbored V179D mutation. The combination of V179D and K103R, conferring intermediate resistance to EFV and NVP, was detected in seven treatment-naive MSM patients.

**Conclusions:**

This study confirmed the expansion CRF65_cpx in China. Furthermore, we found the natural presence of the V179D and K103R/V179D mutations associated with resistance to NNRTIs in HIV-1 CRF65_cpx. Our findings highlight the contribution of polymorphic mutations to drug resistance and underscore the challenges in treating patients harboring CRF65_cpx strains.

## Background

In 2013, a novel HIV type 1 circulating recombinant form (CRF65_cpx) composed of CRF01_AE and subtypes B and C was identified in China [1]. Our previous study demonstrated that CRF65_cpx originated around the year 2000 among heterosexuals in Yunnan Province and subsequently was transmitted to the men who have sex with men (MSM) population in Beijing and Anhui Province about 7 years later [2]. The prevalence of CRF65_cpx among MSM with early infection in Beijing reached 2.3% [2], suggesting an essential contribution of CRF65_cpx to the local HIV-1 epidemic. Recently, sporadic detection of CRF65_cpx strains in MSM who live in Jilin and Hebei provinces might be an indication of CRF65_cpx expansion among the MSM population in China [3, 4]. Of note, CRF65_cpx strains may be misclassified as subtype C strains because of the absence of breakpoints in the *pol* region. The *pol* region is routinely sequenced in the clinical context of genotypic drug resistance testing, and a large body of *pol* sequences are deposited in the Los Alamos HIV Sequence Database (http://www.hiv.lanl.gov/content/index). Therefore, we may be able to identify more CRF65_cpx sequences from the database to investigate its expansion.

Although it seems that combination antiretroviral therapy (ART) is effective against all HIV-1 viral subtypes, there is increasing evidence that HIV-1 genetic diversity can have an impact on drug resistance. On the one hand, natural polymorphisms affect the genetic barrier for the development of drug resistance. The V106M mutation, which confers high-level resistance to efavirenz (EFV) and nevirapine (NVP), is preferentially selected by EFV in subtype C viruses compared with subtype B viruses [5]. Selective acquisition of G190S, which causes higher levels of EFV and NVP resistance than K103N, in HIV-1 subtype A from Russia is another example of a distinct genetic barrier to resistance among different subtypes [6]. On the other hand, the natural presence of drug resistance mutations in some viruses is already being observed. For example, most of the HIV-1 O group viruses are considered to be naturally resistant to nonnucleoside reverse transcriptase inhibitors (NNRTIs) due to the presence of the Y181C mutation [7]. More recently, Zhou et al. reported that CRF01_AE subtype-related resistance to fostemsavir, an attachment inhibitor, appeared to be associated with the natural presence of substitutions S375H and M475I [8].

Drug-resistance mutations (DRMs) can be divided into polymorphic mutations (i.e., mutations occur frequently in viruses not exposed to selective drug pressure) and nonpolymorphic mutations (i.e., mutations do not occur in the absence of therapy) [9]. In general, polymorphic mutations have little or no effect on antiretroviral drug susceptibility. V179D is a common polymorphic mutation that scored by the Stanford HIV Drug Resistance Database as conferring potential low-level resistance to NNRTIs including EFV, NVP, etravirine (ETR), and rilpivirine (RPV) [10, 11]. K103R is also a polymorphic mutation and, alone, has no effect on reduction in phenotypic susceptibility to NNRTIs [12]. However, the combination of V179D and K103R acts synergistically to reduce EFV and NVP susceptibility by about 10- to 15-fold [12], leading to intermediate resistance to EFV and NVP. V179D mutation occurred most frequently in CRF01_AE and subtype F isolates, with a rate of 1.7 and 4.1%, respectively [13]. In 2016, Li et al. reported that the proportion of V179D/E among Shanghai CRF01_AE was 7.1%, which was higher than all other China CRF01_AE [14]. Additionally, V179D/E mutation distributed in 26 networks, suggesting that several independent CRF01_AE strains with V179D/E were involved in ongoing transmission in Shanghai. This observation suggested that the high prevalence of V179D/E was a concern.

In this study, we identified 32 HIV-1 *pol* CRF65_cpx sequences from the Los Alamos HIV Sequence Database, explored the epidemiological relationship of CRF65_cpx strains in different geographic areas and risk groups, and determined the impact of natural polymorphisms of HIV-1 CRF65_cpx on genotypic susceptibility.

## Methods

### Identification of CRF65_cpx sequences from the Los Alamos HIV sequence database

All sequences classified as CRF65_cpx in the Los Alamos HIV Sequence Database were obtained by subtype search at the Sequence Search Interface. To date, there were only 23 CRF65_cpx sequences, including five near full-length genome (NFLG) sequences and 18 partial sequences, deposited in the database. Most of the sequences covered the *pol* region, and the shortest *pol* sequence (accession number: MG706585, HXB2:2260–3311) was subjected to an HIV BLAST search in order to find more CRF65_cpx sequences. The option “number of BLAST matches to display” was set at 100. The outputted 100 sequences were combined with the subtype reference sequences to yield a total dataset of 147 *pol* sequences. Phylogenetic analysis was used to identify all CRF65_cpx sequences from this dataset. MEGA 6.06 [15] software was used to conduct a neighbor-joining phylogenetic tree by using the Kimura 2-parameter model. The confidence of each node in the phylogenetic tree was determined by using the bootstrap method with 1000 replicates. A raw dataset containing 42 CRF65_cpx *pol* sequences was generated.

Subsequently, the raw dataset was carefully scrutinized to remove duplicated sequences using two criteria: (1) If two or more sequences presented the same patient code, we kept only one sequence; and (2) if two or more sequences were retrieved from the same geographic region in the same year, and the genetic distance was zero, only one sequence was kept. After removing duplicated sequences, a refined dataset was generated for further phylogenetic and genotypic resistance analyses.

### Phylogenetic analysis

We performed phylogenetic analysis to clarify the epidemiological relationship of CRF65_cpx strains in China. jModelTest [16] was used to find the best-fit nucleotide substitution model for our dataset. The general time reversible (GTR) model plus a gamma distribution (G4) among site rate heterogeneity and a proportion of invariant sites (I) were chosen as the most appropriate model on the basis of the standard Akaike information criterion. PhyML3.0 [17] was used to estimate a maximum likelihood phylogenetic tree under the above nucleotide substitution model. The topology of the phylogenetic tree was tested by bootstrap analysis with 1000 replicates. The final maximum likelihood tree was visualized by using the program MEGA 6.06.

### Genotypic resistance testing

Genotypic resistance was determined by submitting CRF65_cpx *pol* sequences to the Stanford HIV Drug Resistance Database (https://hivdb.stanford.edu/hivdb/by-sequences/), and DRMs were defined as any mutation with a penalty score.

## Results

### Identification of CRF65_cpx sequences from the Los Alamos HIV sequence database

**Fig. 1:**
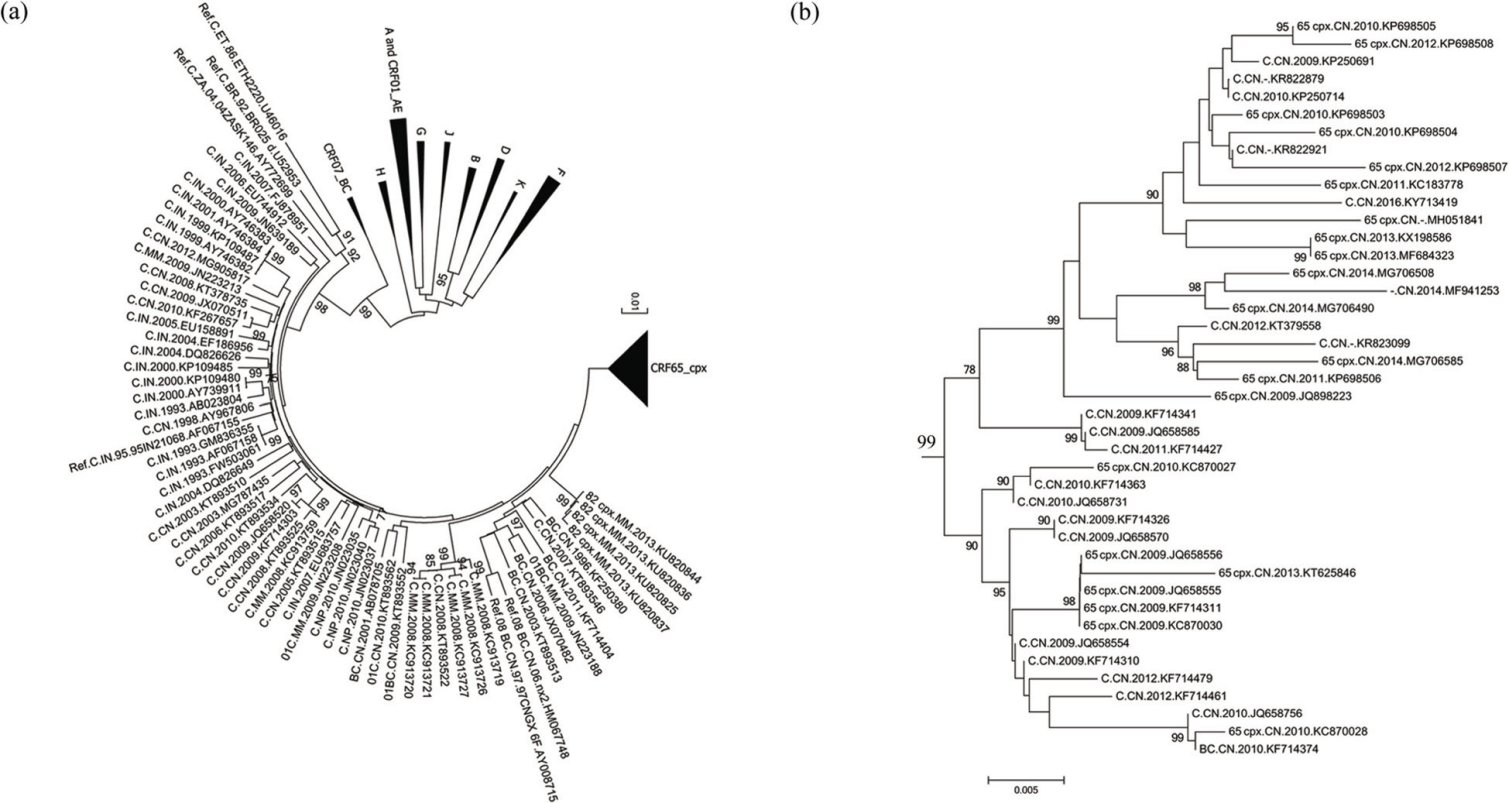
Identification of CRF65_cpx sequences by phylogenetic analysis. **a** The neighbor-joining phylogenetic tree was conducted with 100 HIV BLAST searched *pol* sequences and subtype reference sequences from the Los Alamos HIV Sequence Database. Values on the branches represent the percentage of 1000 bootstrap replicates and bootstrap values over 70% are shown in the tree. The scale bar indicates 1% nucleotide sequence divergence. The identified CRF65_cpx cluster and several subtype reference sequences are compressed to facilitate visualization. **b** Separate display of CRF65_cpx cluster sequences in a neighbor-joining phylogenetic subtree. The scale bar represents 0.5% genetic distance

As shown in the neighbor-joining phylogenetic tree (Fig. 1a), we identified a CRF65_cpx cluster located inside subtype C clade with high bootstrap confidence (99%). Figure 1b shows that some subtype C and 1 B/C recombinant form sequences were intermingled with CRF65_cpx sequences in this cluster, indicating they should be classified as CRF65_cpx sequences. After the removal of duplicated sequences, a total of 32 CRF65_cpx *pol* sequences were obtained. Of note, almost half of the 32 CRF65_cpx sequences (*n* = 32 [46.9%]) were misclassified—14 sequences were misclassified as subtype C and 1 sequence was misclassified as B/C recombinant form (Table 1).

**Table 1:** Demographic and clinical characteristics of 32 patients infected with CRF65_cpx

Accession number	Subtype in the database	Sex	Risk factor	Sampling year	Geographic origin	ART	NNRTI resistance mutations
MG706585	CRF65_cpx	M	MSM	2014	Hebei	Treatment naive	V179D
KP698506	CRF65_cpx	M	NA	2011	NA	Treatment naive	V179D
KT379558	C	M	MSM	2012	Guangdong	Treatment naive	V179D
KR823099	C	M	MSM	2014	Beijing	Treatment naive	V179D
JQ898223	CRF65_cpx	M	IDU	2009	Yunnan	Treatment experienced	V179D, Y181C, H221Y
KR822879	C	M	MSM	2010	Beijing	Treatment naive	V179D
MG706490	CRF65_cpx	M	MSM	2014	Hebei	Treatment naive	V179D
KP250691	C	M	MSM	2009	Beijing	Treatment naive	V179D
MG706508	CRF65_cpx	M	MSM	2014	Hebei	Treatment naive	V179D
KP698505	CRF65_cpx	M	MSM	2010	Beijing	Treatment naive	V179D
KP698503	CRF65_cpx	M	MSM	2010	Beijing	Treatment naive	V179D
KX198586	CRF65_cpx	M	MSM	2013	Hebei	Treatment naive	K103R, V179D
KF714341	C	F	HET	2009	Yunnan	Treatment naive	V179D
KP698508	CRF65_cpx	M	MSM	2012	Beijing	Treatment naive	K103R, V179D
KP698504	CRF65_cpx	M	MSM	2010	Beijing	Treatment naive	K103R, V179D
KF714427	C	M	HET	2011	Yunnan	Treatment naive	V179D
KR822921	C	M	MSM	2011	Beijing	Treatment naive	K103R, V179D
KC183778	CRF65_cpx	M	MSM	2011	Anhui	Treatment naive	K103R, V179D
KF714326	C	M	HET	2009	Yunnan	Treatment naive	V179D
KP698507	CRF65_cpx	M	MSM	2012	Beijing	Treatment naive	V179D
KY713419	C	M	MSM	2016	Beijing	Treatment naive	K103R, V179D
KF714479	C	F	HET	2012	Yunnan	Treatment naive	V179D
MH051841	CRF65_cpx	M	MSM	2015	Jilin	Treatment naive	K103R, V179D
KF714310	C	F	HET	2009	Yunnan	Treatment naive	V179D
KF714461	C	F	HET	2012	Yunnan	Treatment naive	E138A, V179D
KC870030	CRF65_cpx	M	HET	2009	Yunnan	Treatment naive	V179D
KC870027	CRF65_cpx	F	HET	2010	Yunnan	Treatment naive	V179D
KF714363	C	F	HET	2010	Yunnan	Treatment naive	V179D
MF941253	C	NA	NA	2014	Heilongjiang	Treatment naive	K103R, V179E
KT625846	CRF65_cpx	NA	NA	2013	Yunnan	Treatment naive	V179D
KC870028	CRF65_cpx	F	HET	2010	Yunnan	Treatment naive	V179D
KF714374	BC	F	HET	2010	Yunnan	Treatment naive	V179D

*ART* antiretroviral therapy, *M* male, *F* female, *HET* heterosexual, *IDU* intravenous drug user, *MSM* men who have sex with men, *NA* not available

### Expansion of CRF65_cpx in China

**Fig. 2:**
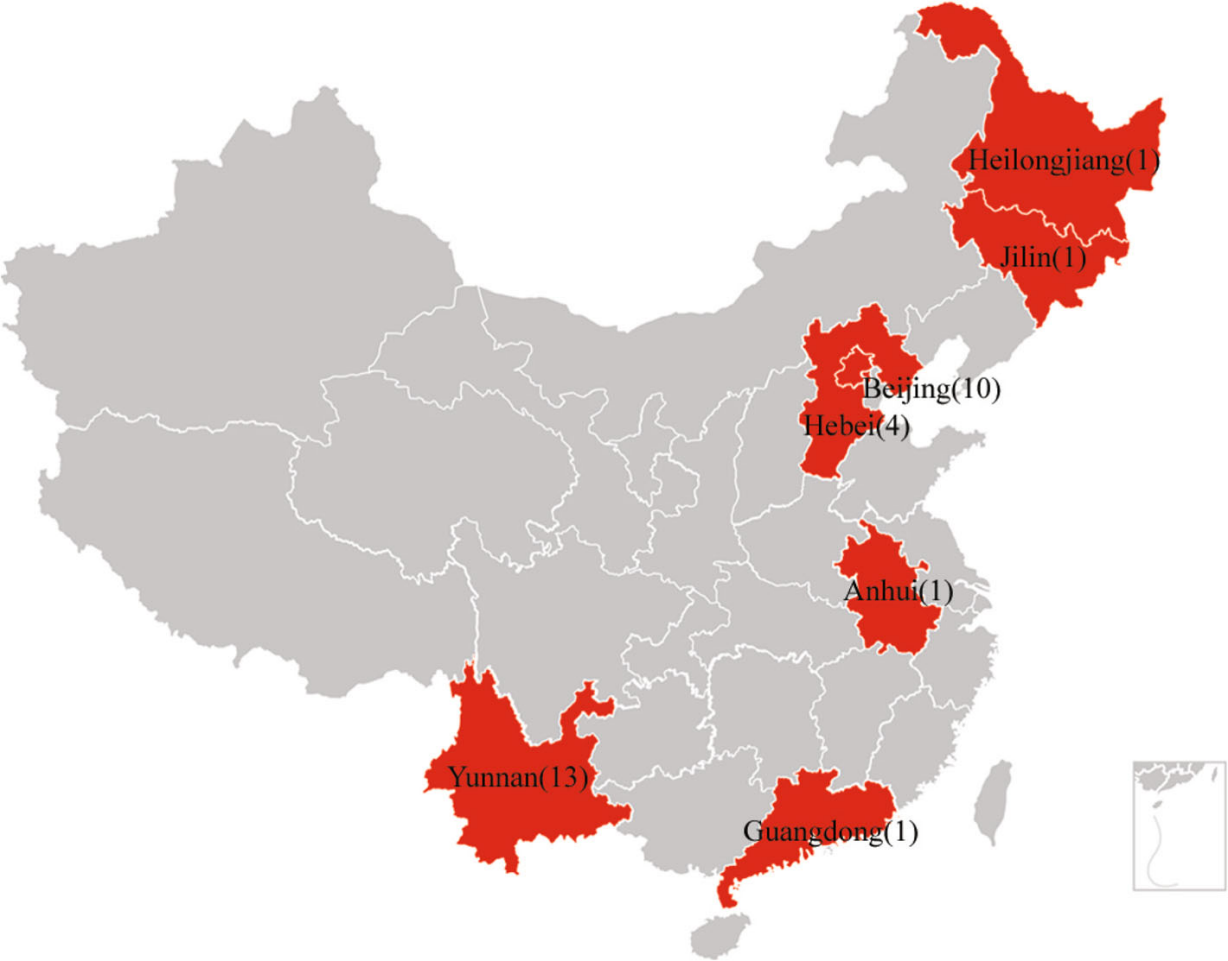
Geographic distribution of HIV-1 CRF65_cpx strains identified in this study. On the map of China, the provinces where CRF65_cpx was detected were painted in red color and marked with the number of CRF65_cpx strains

Figure 2 illustrates the geographic distribution of CRF65_cpx in China. In the present study, we found that the CRF65_cpx strains were detected in seven provinces that are geographically far from each other: southern provinces (Yunnan and Guangdong), northern provinces (Jilin and Heilongjiang), eastern province (Anhui), and central provinces (Beijing and Hebei). Yunnan Province and Beijing were the two areas where the majority (71.9%, 23/32) of CRF65_cpx strains were detected. Hebei, Anhui, Guangdong, Jilin, and Heilongjiang provinces collectively harbored one-quarter (25%, 8/32) of CRF65_cpx strains, and the geographic origin of one strain (accession number: KP698506) was not reported. Although the number of CRF65_cpx strains detected in these five provinces was small, the strains were all detected in recent years, which provided evidence for onward transmission of CRF65_cpx. The detection of CRF65_cpx strains in an increasing number of provinces suggested the rapid expansion of this variant in China.

**Fig. 3:**
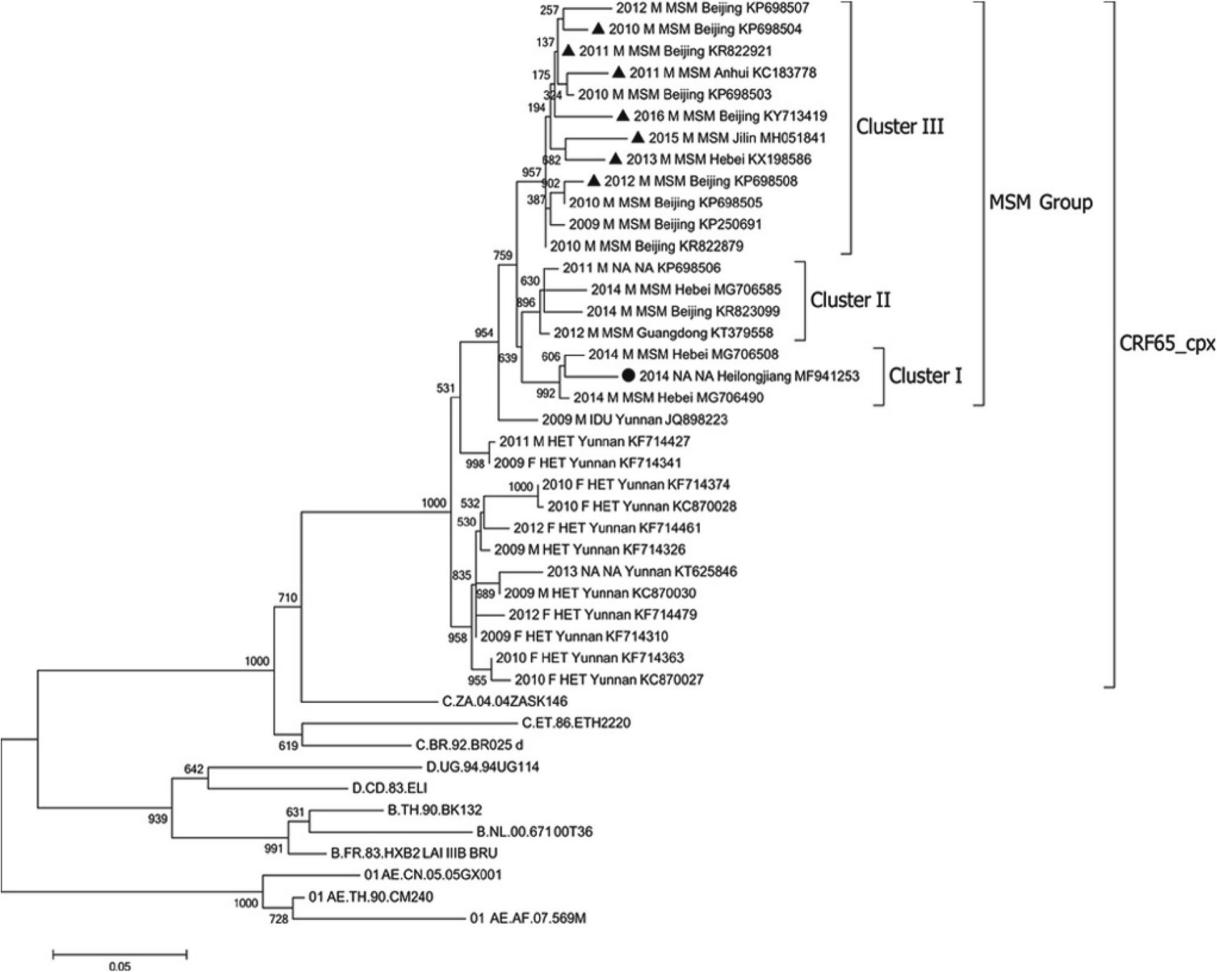
Phylogenetic maximum likelihood tree of CRF65_CPX. Maximum likelihood phylogeny was estimated with all CRF65_CPX sequences and several subtype reference sequences by PhyML 3.0 software. Subtype reference sequences were retrieved from the Los Alamos HIV Sequence Database. The scale bar represents 5% genetic distance. CRF65_CPX sequences were sequentially labeled with sampling year, sex, risk group, geographic origin and accession number. Sequences are indicated by symbols corresponding to their NNRTI resistance mutation patterns: K103R + V179D (triangle), K103R + V179E (circle)

As shown in the ML tree (Fig. 3), all CRF65_cpx sequences formed a monophyletic cluster with a high bootstrap value of 1000. CRF65_cpx sequences from Yunnan Province were located near the root of this cluster, and the remainder of CRF65_cpx sequences from the other six provinces formed an MSM group and positioned inside the CRF65_cpx cluster. To our surprise, Yunnan CRF65_cpx sequences were mainly derived from a heterosexual risk group (84.6%, 11/13), whereas the CRF65_cpx sequences in other provinces were almost exclusively derived from an MSM population (94.4%, 17/18). These results suggested that CRF65_cpx initially originated among heterosexuals in Yunnan Province and subsequently spread to MSM in other provinces, which was in line with our previous study observations [2]. The ML phylogenetic tree also showed that all MSM group sequences (*n* = 19) were classified into three different clusters (Fig. 3). Cluster I included two Hebei sequences and one Heilongjiang sequence. Cluster II involved Hebei, Beijing, and Guangdong sequences and one sequence whose geographic origin was not available. We found one larger cluster (cluster III) that grouped 12 sequences from four provinces: nine from Beijing, one from Hebei, one from Jilin, and one from Anhui. The inclusion of sequences from different provinces in all three MSM clusters illustrated the active transmission of CRF65_cpx among MSM, regardless of a large geographic distance.

### Natural presence of the V179D and K103R/V179D mutations in CRF65_cpx strains

Among the 32 patients harboring CRF65_cpx strains, the great majority (96.9%, 31/32) were treatment naive and the remaining one patient was treatment experienced. We surprisingly found NNRTI-resistance mutations were present in all of the 32 patients. With the exception of one patient who had NNRTI-resistance mutation V179E, the other 31 patients harbored V179D mutation, which resulted in the natural presence of V179D in CRF65_cpx strains (Table 1). All V179D mutations were encoded by codon GAT, whereas the V179E mutation was encoded by codon GAA. A single T-to-A transversion at the third position of codon 179 in CRF65_cpx may be responsible for the emergence of V179E mutation in one patient. Obviously, V179D mutation was a signature mutation in CRF65_cpx patients due to a strong founder effect.

Variants emerging at RT position 179 were highly heterogeneous and most often accompanied by other DRMs. V179D occurred alone in 22 patients, accounting for nearly 70% of all patients with CRF65_cpx. Surprisingly, the combination of V179D and K103R, conferring intermediate resistance to EFV and NVP, was detected in seven treatment-naive patients, also indicating the natural presence of K103R/V179D in CRF65_cpx strains (Table 1). We also found that two polymorphic accessory mutations, K103R and V179E, occurred simultaneously in one patient. In addition, V179D mutation was accompanied by E138A mutation in one patient, and these two mutations resulted in low-level resistance to ETR and RPV. However, the effects of combinations of K103R plus V179E and E138A plus V179D on NNRTI susceptibility are still not clear, which suggested that these two mutation patterns deserve further investigation. V179D, Y181C, and H221Y were detected in the one treatment-experienced patient.

We next sought to investigate the distribution of the K103R/V179D mutation in different risk groups and geographic areas. As shown in Fig. 3, none of Yunnan sequences harbored the K103R/V179D mutation, regardless of the risk group. Of note, all seven CRF65_cpx sequences harboring K103R/V179D mutation were exclusively present in MSM cluster III. Moreover, sequences harboring K103R/V179D mutation accounted for more than half (58.3%, 7/12) of all cluster III sequences, demonstrating the natural presence of the K103R/V179D mutation in this larger MSM cluster. With respect to geographic distribution, K103R/V179D mutation was detected in four provinces: four patients from Beijing, one patient from Hebei, one patient from Jilin, and one patient from Anhui.

## Discussion

The rapid expansion of new HIV-1 subtypes or CRFs poses new challenges in clinical management, diagnostic testing, and vaccine design. To investigate the expansion of HIV-1 genotypes, it is common practice to use molecular biological methods to identify strains in a certain number of samples [18, 19]. However, when the prevalence of a particular genotype is extremely low, a very large number of samples is required, making the investigation difficult. CRF65_cpx was such an example with very low prevalence [2]. However, given the absence of breakpoints in CRF65_cpx *pol* region, the possibility that some CRF65_cpx strains are misclassified cannot be excluded. In the present study, we used the BLAST search program, followed by phylogenetic analysis, and we identified additional 15 CRF65_cpx *pol* sequences from the Los Alamos HIV Sequence Database. Similarly, we believe that this method can also be used to investigate other HIV-1 genotypes with low prevalence.

It has been demonstrated that the V179D mutation, conferring potential low-level resistance to NNRTIs, occurred most frequently in CRF01_AE and subtype F viruses, at a rate not greater than 5% [13]. Previously, no study reported the presence of V179D in almost all strains of a subtype or CRF. Our study showed that almost all CRF65_cpx strains harbored V179D mutation, and V179D was considered as a signature mutation in CRF65_cpx strains. Regarding the origin of V179D in CRF65 strain, we think there are two possible reasons. Firstly, one possible reason is that a subtype C parental strain containing V179D mutation was involved in the recombination event, which resulted in the presence of V179D in the founder CRF65_cpx strain. Subsequently, the V179D was transmitted to other patients along with the spread of CRF65_cpx. Secondly, considering that V179D is a polymorphic mutation, theoretically there is another reason. This mutation might emerge in the founder strain of CRF65_cpx during a short window period after the generation of CRF65_cpx and before its onward transmission. In other words, the sequence of events was as follows: generation of CRF65_cpx, acquisition of V179D mutation, and propagation of CRF65_cpx.

Although a single polymorphic mutation does not result in a significant decrease in susceptibility, there is evidence that polymorphic mutations can have an important effect on drug resistance. It is known that the combination of two polymorphic NNRTI-associated mutations can increase drug resistance level. For example, combinations of K103R plus V179D and V106I plus V179D confer intermediate and low reduction in EFV and NVP susceptibility, respectively [12, 20]. K103R/V179D mutation was not frequently detected because of the low occurrence of both K103R and V179D in untreated persons [12]. In a recent study, only three isolates had the mutations K103R/V179D in a dataset containing more than 1700 isolates, which showed that the prevalence of K103R/V179D was extremely low [11]. However, among the 32 patients harboring CRF65_cpx strains, seven patients had the K103R/V179D mutation, illustrating the surprisingly high prevalence of K103R/V179D in CRF65_cpx strains (21.9%, 7/32). More importantly, these seven sequences were exclusively distributed among MSM group and MSM cluster III, which resulted in a much higher prevalence of K103R/V179D (Fig. 3). Obviously, a founder effect resulted in the presence of V179D in CRF65_cpx, and K103R subsequently emerged in some strains after CRF65_cpx spread to MSM population.

Except for K103R plus V179D, two new combinations of K103R plus V179E and E138A plus V179D were identified in two drug-naive patients. The combination of K103R and V179E was recommended by the Stanford HIV Drug Resistance Database as likely to reduce NVP and EFV susceptibility as the combination of K103R and V179D. E138A is also a polymorphic mutation and reduces ETR and RPV susceptibility by about 2-fold [21]. It was reported that E138A is more prevalent in subtype C than in subtype B in different databases [22]. E138A could have an impact on treatment or prevention strategies that include ETR or RPV in geographic areas where subtype C infection is dominating. Interestingly, Giannini and colleagues found that E138A can contribute to reduced response to ETR through a decreased genetic barrier to resistance, indicating the distinct mechanism of E138A resistance to ETR [23]. Together, the impact of K103R/V179E and E138A/V179D on both NNRTI susceptibility and virologic outcome in patients deserves investigation.

The recent epidemiology of HIV-1 infection among MSM in China has been characterized by a wide distribution of multiple HIV-1 genotypes. Unprotected sex, having multiple sexual partners, and limited knowledge of HIV infection make Chinese MSM significantly more vulnerable to infection [24]. Importantly, the high mobility presented by the MSM population facilitated the rapid dissemination of diverse forms of HIV-1 across China. It seems that one subtype or CRF circulating in Chinese MSM can easily break through geographical barriers. For example, a recent nationwide survey revealed that two CRF01_AE lineages and one CRF07_BC lineage were responsible for the recent upsurge of the AIDS epidemic among MSM, and these three HIV-1 variants were spread widely among MSM throughout China [25]. Although CRF55_01B was a recently identified CRF among MSM, this variant also was disseminated widely among MSM in some big cities of China [26]. Consequently, we can forecast that CRF65_cpx strains will be detected among MSM in an increasingly number of geographic regions in the near future. Another important observation was that K103R/V179D mutations were exclusively present in the MSM population and distributed in different provinces. The transmission of CRF65_cpx strains containing K103R/V179D mutation to persons who are treatment naive can compromise the effectiveness of treatment and limit antiretroviral regimen options [9]. Therefore, molecular surveillance of CRF65_cpx strains, particularly among MSM population, is of great importance.

## Conclusions

In summary, we confirmed the expansion of HIV-1 CRF65_cpx in China and, surprisingly, found the natural presence of the V179D and K103R/V179D mutations in HIV-1 CRF65_cpx. Our findings highlight the contribution of polymorphic mutations to drug resistance and underscore the challenges in treating patients harboring CRF65_cpx strains.

## Data Availability

The datasets used and/or analyzed during the current study are available from the corresponding author on reasonable request.

## Abbreviations

MSM: Men who have sex with men
ART: Antiretroviral therapy
EFV: Efavirenz
NVP: Nevirapine
NNRTIs: Nonnucleoside reverse transcriptase inhibitors
DRMs: Drug-resistance mutations
ETR: Etravirine
RPV: Rilpivirine
NFLG: Near full-length genome
